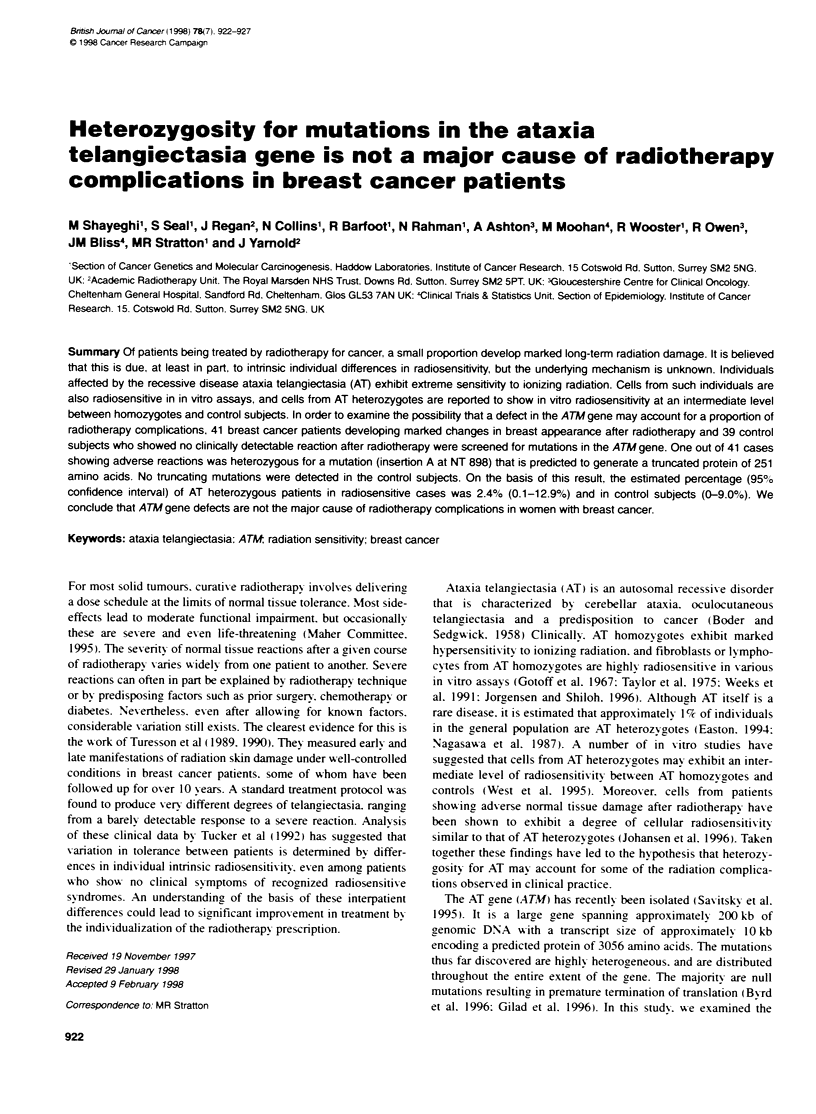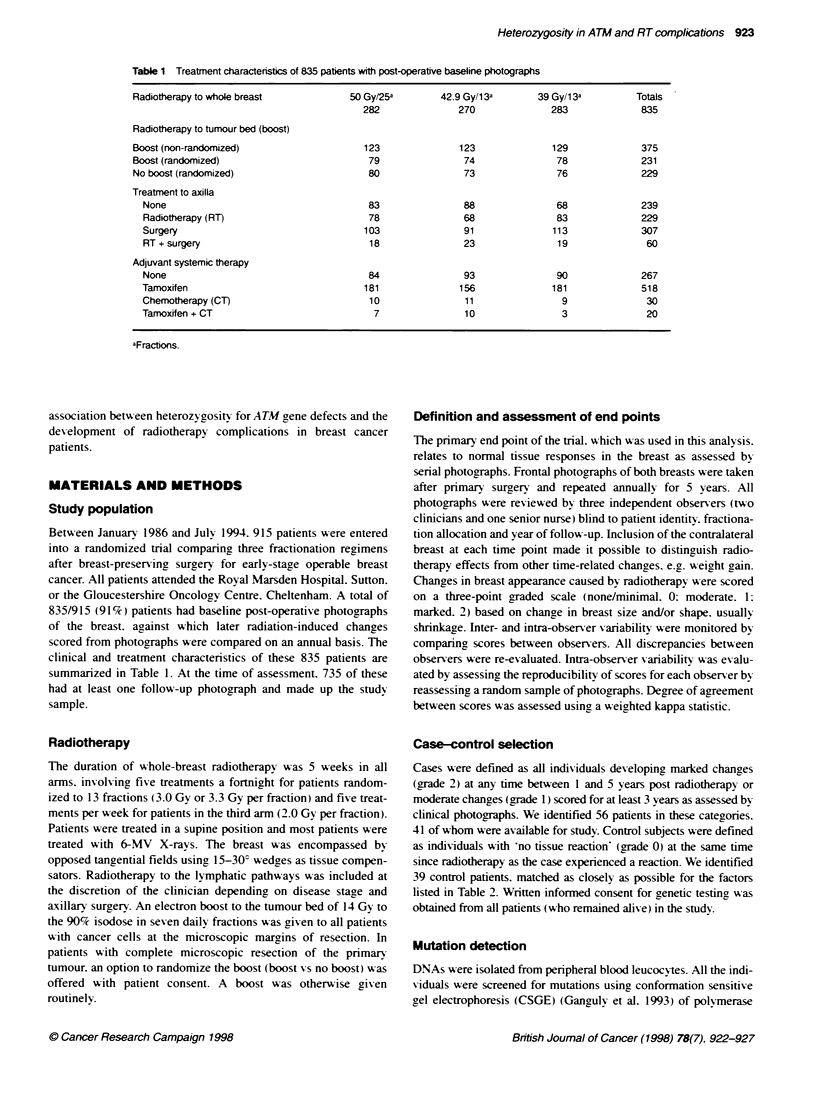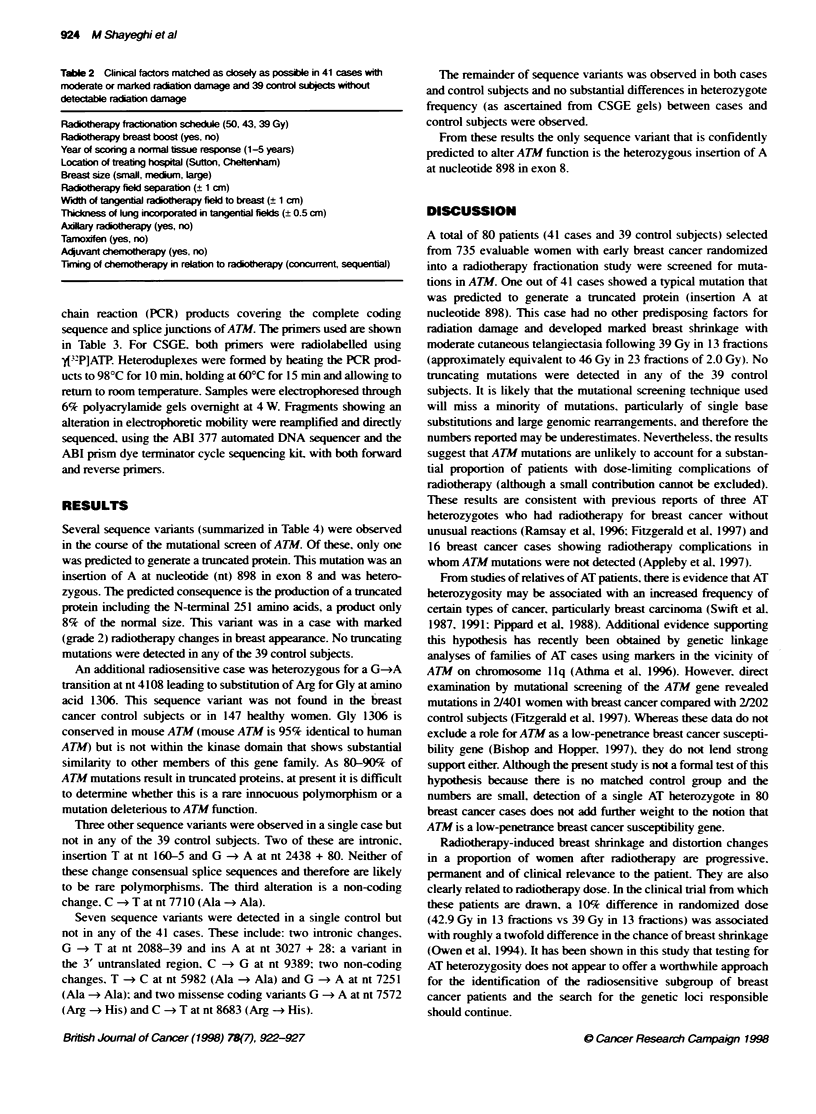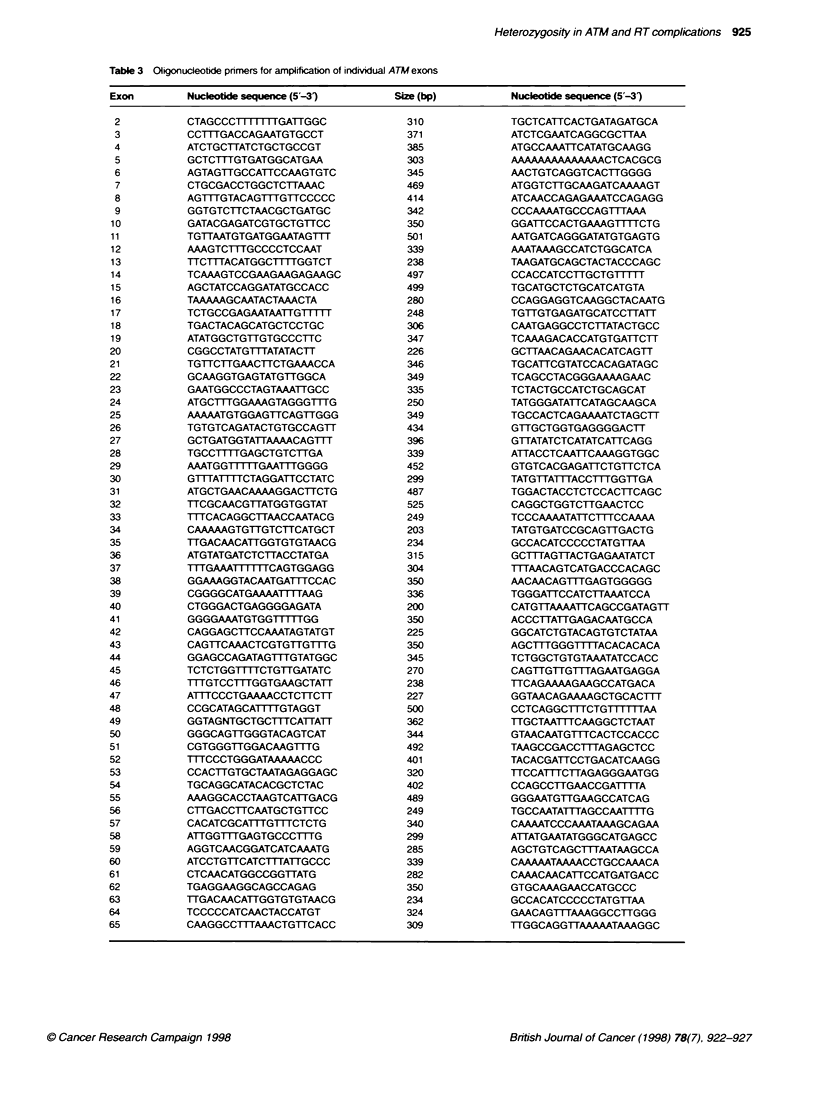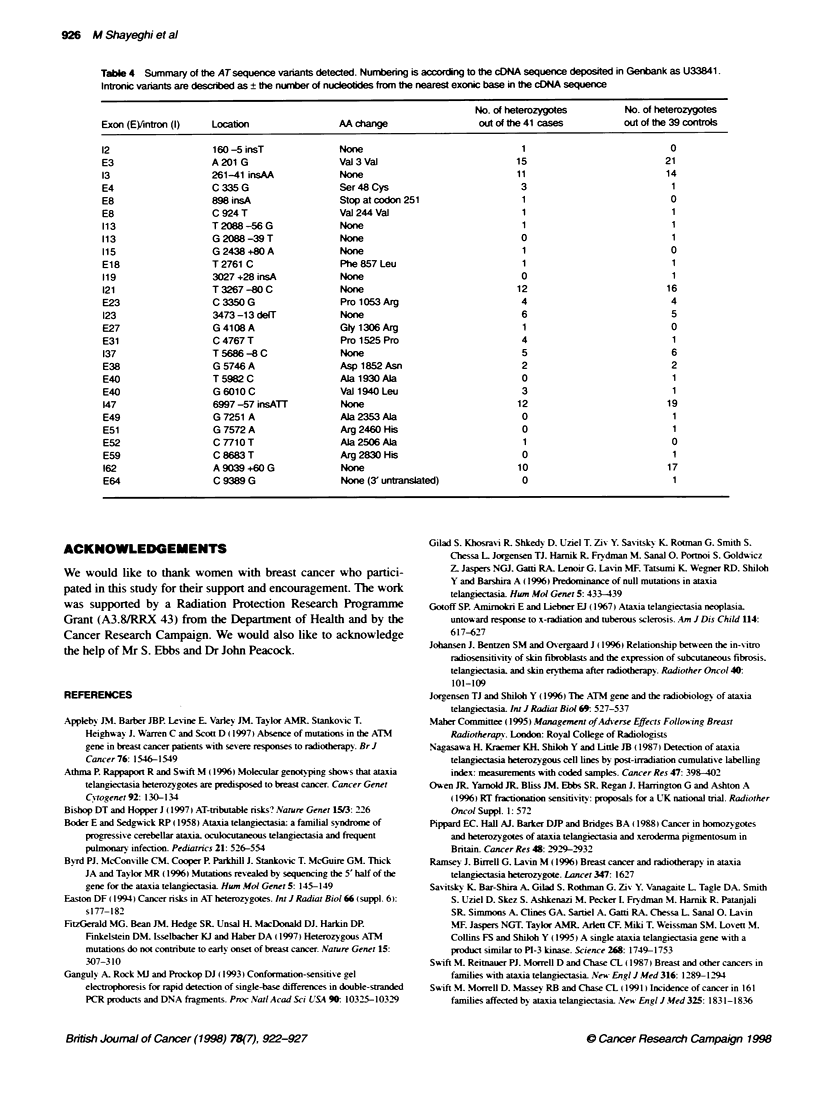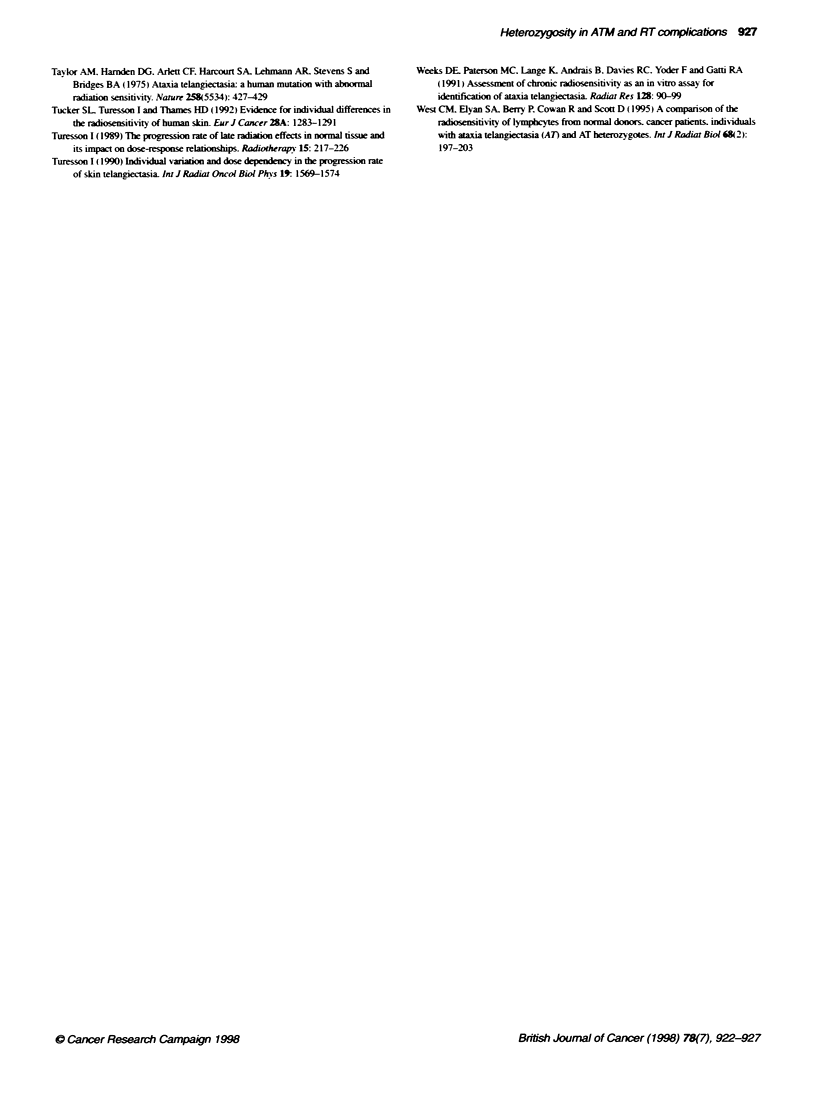# Heterozygosity for mutations in the ataxia telangiectasia gene is not a major cause of radiotherapy complications in breast cancer patients.

**DOI:** 10.1038/bjc.1998.602

**Published:** 1998-10

**Authors:** M. Shayeghi, S. Seal, J. Regan, N. Collins, R. Barfoot, N. Rahman, A. Ashton, M. Moohan, R. Wooster, R. Owen, J. M. Bliss, M. R. Stratton, J. Yarnold

**Affiliations:** Section of Cancer Genetics and Molecular Carcinogenesis, Haddow Laboratories, Institute of Cancer Research, Sutton, Surrey, UK.

## Abstract

Of patients being treated by radiotherapy for cancer, a small proportion develop marked long-term radiation damage. It is believed that this is due, at least in part, to intrinsic individual differences in radiosensitivity, but the underlying mechanism is unknown. Individuals affected by the recessive disease ataxia telangiectasia (AT) exhibit extreme sensitivity to ionizing radiation. Cells from such individuals are also radiosensitive in in vitro assays, and cells from AT heterozygotes are reported to show in vitro radiosensitivity at an intermediate level between homozygotes and control subjects. In order to examine the possibility that a defect in the ATM gene may account for a proportion of radiotherapy complications, 41 breast cancer patients developing marked changes in breast appearance after radiotherapy and 39 control subjects who showed no clinically detectable reaction after radiotherapy were screened for mutations in the ATM gene. One out of 41 cases showing adverse reactions was heterozygous for a mutation (insertion A at NT 898) that is predicted to generate a truncated protein of 251 amino acids. No truncating mutations were detected in the control subjects. On the basis of this result, the estimated percentage (95% confidence interval) of AT heterozygous patients in radiosensitive cases was 2.4% (0.1-12.9%) and in control subjects (0-9.0%). We conclude that ATM gene defects are not the major cause of radiotherapy complications in women with breast cancer.


British Journal of Cancer (1998) 78(7), 922–927
© 1998 Cancer Research Campaign

### Heterozygosity for mutations in the ataxia telangiectasia gene is not a major cause of radiotherapy complications in breast cancer patients

**M Shayeghi[1], S Seal[1], J Regan[2], N Collins[1], R Barfoot[1], N Rahman[1], A Ashton[3], M Moohan[4], R Wooster[1], R Owen[3], JM Bliss[4], MR Stratton[1] and J Yarnold[2]**

[1]Section of Cancer Genetics and Molecular Carcinogenesis, Haddow Laboratories, Institute of Cancer Research, 15 Cotswold Rd, Sutton, Surrey SM2 5NG, UK; [2]Academic Radiotherapy Unit, The Royal Marsden NHS Trust, Downs Rd, Sutton, Surrey SM2 5PT, UK; [3]Gloucestershire Centre for Clinical Oncology, Cheltenham General Hospital, Sandford Rd, Cheltenham, Glos GL53 7AN UK; [4]Clinical Trials & Statistics Unit, Section of Epidemiology, Institute of Cancer Research, 15, Cotswold Rd, Sutton, Surrey SM2 5NG, UK

**Summary** Of patients being treated by radiotherapy for cancer, a small proportion develop marked long-term radiation damage. It is believed that this is due, at least in part, to intrinsic individual differences in radiosensitivity, but the underlying mechanism is unknown. Individuals affected by the recessive disease ataxia telangiectasia (AT) exhibit extreme sensitivity to ionizing radiation. Cells from such individuals are also radiosensitive in in vitro assays, and cells from AT heterozygotes are reported to show in vitro radiosensitivity at an intermediate level between homozygotes and control subjects. In order to examine the possibility that a defect in the *ATM* gene may account for a proportion of radiotherapy complications, 41 breast cancer patients developing marked changes in breast appearance after radiotherapy and 39 control subjects who showed no clinically detectable reaction after radiotherapy were screened for mutations in the *ATM* gene. One out of 41 cases showing adverse reactions was heterozygous for a mutation (insertion A at NT 898) that is predicted to generate a truncated protein of 251 amino acids. No truncating mutations were detected in the control subjects. On the basis of this result, the estimated percentage (95% confidence interval) of AT heterozygous patients in radiosensitive cases was 2.4% (0.1–12.9%) and in control subjects (0–9.0%). We conclude that *ATM* gene defects are not the major cause of radiotherapy complications in women with breast cancer.

**Keywords:** ataxia telangiectasia; *ATM*; radiation sensitivity; breast cancer

For most solid tumours, curative radiotherapy involves delivering a dose schedule at the limits of normal tissue tolerance. Most side-effects lead to moderate functional impairment, but occasionally these are severe and even life-threatening (Maher Committee, 1995). The severity of normal tissue reactions after a given course of radiotherapy varies widely from one patient to another. Severe reactions can often in part be explained by radiotherapy technique or by predisposing factors such as prior surgery, chemotherapy or diabetes. Nevertheless, even after allowing for known factors, considerable variation still exists. The clearest evidence for this is the work of Turesson et al (1989, 1990). They measured early and late manifestations of radiation skin damage under well-controlled conditions in breast cancer patients, some of whom have been followed up for over 10 years. A standard treatment protocol was found to produce very different degrees of telangiectasia, ranging from a barely detectable response to a severe reaction. Analysis of these clinical data by Tucker et al (1992) has suggested that variation in tolerance between patients is determined by differences in individual intrinsic radiosensitivity, even among patients who show no clinical symptoms of recognized radiosensitive syndromes. An understanding of the basis of these interpatient differences could lead to significant improvement in treatment by the individualization of the radiotherapy prescription.

*Received 19 November 1997*
*Revised 29 January 1998*
*Accepted 9 February 1998*

*Correspondence to:* MR Stratton

Ataxia telangiectasia (AT) is an autosomal recessive disorder that is characterized by cerebellar ataxia, oculocutaneous telangiectasia and a predisposition to cancer (Boder and Sedgwick, 1958) Clinically, AT homozygotes exhibit marked hypersensitivity to ionizing radiation, and fibroblasts or lymphocytes from AT homozygotes are highly radiosensitive in various in vitro assays (Gotoff et al, 1967; Taylor et al, 1975; Weeks et al, 1991; Jorgensen and Shiloh, 1996). Although AT itself is a rare disease, it is estimated that approximately 1% of individuals in the general population are AT heterozygotes (Easton, 1994; Nagasawa et al, 1987). A number of in vitro studies have suggested that cells from AT heterozygotes may exhibit an intermediate level of radiosensitivity between AT homozygotes and controls (West et al, 1995). Moreover, cells from patients showing adverse normal tissue damage after radiotherapy have been shown to exhibit a degree of cellular radiosensitivity similar to that of AT heterozygotes (Johansen et al, 1996). Taken together these findings have led to the hypothesis that heterozygosity for AT may account for some of the radiation complications observed in clinical practice.

The AT gene (*ATM*) has recently been isolated (Savitsky et al, 1995). It is a large gene spanning approximately 200 kb of genomic DNA with a transcript size of approximately 10 kb encoding a predicted protein of 3056 amino acids. The mutations thus far discovered are highly heterogeneous, and are distributed throughout the entire extent of the gene. The majority are null mutations resulting in premature termination of translation (Byrd et al, 1996; Gilad et al, 1996). In this study, we examined the

**Table 1** Treatment characteristics of 835 patients with post-operative baseline photographs

| Radiotherapy to whole breast | 50 Gy/25[a] 282 | 42.9 Gy/13[a] 270 | 39 Gy/13[a] 283 | Totals 835 |
|---|---|---|---|---|
| Radiotherapy to tumour bed (boost) | | | | |
| Boost (non-randomized) | 123 | 123 | 129 | 375 |
| Boost (randomized) | 79 | 74 | 78 | 231 |
| No boost (randomized) | 80 | 73 | 76 | 229 |
| Treatment to axilla | | | | |
| None | 83 | 88 | 68 | 239 |
| Radiotherapy (RT) | 78 | 68 | 83 | 229 |
| Surgery | 103 | 91 | 113 | 307 |
| RT + surgery | 18 | 23 | 19 | 60 |
| Adjuvant systemic therapy | | | | |
| None | 84 | 93 | 90 | 267 |
| Tamoxifen | 181 | 156 | 181 | 518 |
| Chemotherapy (CT) | 10 | 11 | 9 | 30 |
| Tamoxifen + CT | 7 | 10 | 3 | 20 |

[a]Fractions.

association between heterozygosity for *ATM* gene defects and the development of radiotherapy complications in breast cancer patients.

### MATERIALS AND METHODS

### Study population

Between January 1986 and July 1994, 915 patients were entered into a randomized trial comparing three fractionation regimens after breast-preserving surgery for early-stage operable breast cancer. All patients attended the Royal Marsden Hospital, Sutton, or the Gloucestershire Oncology Centre, Cheltenham. A total of 835/915 (91%) patients had baseline post-operative photographs of the breast, against which later radiation-induced changes scored from photographs were compared on an annual basis. The clinical and treatment characteristics of these 835 patients are summarized in Table 1. At the time of assessment, 735 of these had at least one follow-up photograph and made up the study sample.

### Radiotherapy

The duration of whole-breast radiotherapy was 5 weeks in all arms, involving five treatments a fortnight for patients randomized to 13 fractions (3.0 Gy or 3.3 Gy per fraction) and five treatments per week for patients in the third arm (2.0 Gy per fraction). Patients were treated in a supine position and most patients were treated with 6-MV X-rays. The breast was encompassed by opposed tangential fields using 15–30° wedges as tissue compensators. Radiotherapy to the lymphatic pathways was included at the discretion of the clinician depending on disease stage and axillary surgery. An electron boost to the tumour bed of 14 Gy to the 90% isodose in seven daily fractions was given to all patients with cancer cells at the microscopic margins of resection. In patients with complete microscopic resection of the primary tumour, an option to randomize the boost (boost vs no boost) was offered with patient consent. A boost was otherwise given routinely.

### Definition and assessment of end points

The primary end point of the trial, which was used in this analysis, relates to normal tissue responses in the breast as assessed by serial photographs. Frontal photographs of both breasts were taken after primary surgery and repeated annually for 5 years. All photographs were reviewed by three independent observers (two clinicians and one senior nurse) blind to patient identity, fractionation allocation and year of follow-up. Inclusion of the contralateral breast at each time point made it possible to distinguish radiotherapy effects from other time-related changes, e.g. weight gain. Changes in breast appearance caused by radiotherapy were scored on a three-point graded scale (none/minimal, 0; moderate, 1; marked, 2) based on change in breast size and/or shape, usually shrinkage. Inter- and intra-observer variability were monitored by comparing scores between observers. All discrepancies between observers were re-evaluated. Intra-observer variability was evaluated by assessing the reproducibility of scores for each observer by reassessing a random sample of photographs. Degree of agreement between scores was assessed using a weighted kappa statistic.

### Case–control selection

Cases were defined as all individuals developing marked changes (grade 2) at any time between 1 and 5 years post radiotherapy or moderate changes (grade 1) scored for at least 3 years as assessed by clinical photographs. We identified 56 patients in these categories, 41 of whom were available for study. Control subjects were defined as individuals with 'no tissue reaction' (grade 0) at the same time since radiotherapy as the case experienced a reaction. We identified 39 control patients, matched as closely as possible for the factors listed in Table 2. Written informed consent for genetic testing was obtained from all patients (who remained alive) in the study.

### Mutation detection

DNAs were isolated from peripheral blood leucocytes. All the individuals were screened for mutations using conformation sensitive gel electrophoresis (CSGE) (Ganguly et al, 1993) of polymerase

© Cancer Research Campaign 1998

Table 2 Clinical factors matched as closely as possible in 41 cases with moderate or marked radiation damage and 39 control subjects without detectable radiation damage

| |
|---|
| Radiotherapy fractionation schedule (50, 43, 39 Gy) |
| Radiotherapy breast boost (yes, no) |
| Year of scoring a normal tissue response (1–5 years) |
| Location of treating hospital (Sutton, Cheltenham) |
| Breast size (small, medium, large) |
| Radiotherapy field separation (± 1 cm) |
| Width of tangential radiotherapy field to breast (± 1 cm) |
| Thickness of lung incorporated in tangential fields (± 0.5 cm) |
| Axillary radiotherapy (yes, no) |
| Tamoxifen (yes, no) |
| Adjuvant chemotherapy (yes, no) |
| Timing of chemotherapy in relation to radiotherapy (concurrent, sequential) |

chain reaction (PCR) products covering the complete coding sequence and splice junctions of *ATM*. The primers used are shown in Table 3. For CSGE, both primers were radiolabelled using $\gamma[^{32}P]$ATP. Heteroduplexes were formed by heating the PCR products to 98°C for 10 min, holding at 60°C for 15 min and allowing to return to room temperature. Samples were electrophoresed through 6% polyacrylamide gels overnight at 4 W. Fragments showing an alteration in electrophoretic mobility were reamplified and directly sequenced, using the ABI 377 automated DNA sequencer and the ABI prism dye terminator cycle sequencing kit, with both forward and reverse primers.

### RESULTS

Several sequence variants (summarized in Table 4) were observed in the course of the mutational screen of *ATM*. Of these, only one was predicted to generate a truncated protein. This mutation was an insertion of A at nucleotide (nt) 898 in exon 8 and was heterozygous. The predicted consequence is the production of a truncated protein including the N-terminal 251 amino acids, a product only 8% of the normal size. This variant was in a case with marked (grade 2) radiotherapy changes in breast appearance. No truncating mutations were detected in any of the 39 control subjects.

An additional radiosensitive case was heterozygous for a G→A transition at nt 4108 leading to substitution of Arg for Gly at amino acid 1306. This sequence variant was not found in the breast cancer control subjects or in 147 healthy women. Gly 1306 is conserved in mouse *ATM* (mouse *ATM* is 95% identical to human *ATM*) but is not within the kinase domain that shows substantial similarity to other members of this gene family. As 80–90% of *ATM* mutations result in truncated proteins, at present it is difficult to determine whether this is a rare innocuous polymorphism or a mutation deleterious to *ATM* function.

Three other sequence variants were observed in a single case but not in any of the 39 control subjects. Two of these are intronic, insertion T at nt 160–5 and G → A at nt 2438 + 80. Neither of these change consensual splice sequences and therefore are likely to be rare polymorphisms. The third alteration is a non-coding change, C → T at nt 7710 (Ala → Ala).

Seven sequence variants were detected in a single control but not in any of the 41 cases. These include: two intronic changes, G → T at nt 2088–39 and ins A at nt 3027 + 28; a variant in the 3′ untranslated region, C → G at nt 9389; two non-coding changes, T → C at nt 5982 (Ala → Ala) and G → A at nt 7251 (Ala → Ala); and two missense coding variants G → A at nt 7572 (Arg → His) and C → T at nt 8683 (Arg → His).

The remainder of sequence variants was observed in both cases and control subjects and no substantial differences in heterozygote frequency (as ascertained from CSGE gels) between cases and control subjects were observed.

From these results the only sequence variant that is confidently predicted to alter *ATM* function is the heterozygous insertion of A at nucleotide 898 in exon 8.

### DISCUSSION

A total of 80 patients (41 cases and 39 control subjects) selected from 735 evaluable women with early breast cancer randomized into a radiotherapy fractionation study were screened for mutations in *ATM*. One out of 41 cases showed a typical mutation that was predicted to generate a truncated protein (insertion A at nucleotide 898). This case had no other predisposing factors for radiation damage and developed marked breast shrinkage with moderate cutaneous telangiectasia following 39 Gy in 13 fractions (approximately equivalent to 46 Gy in 23 fractions of 2.0 Gy). No truncating mutations were detected in any of the 39 control subjects. It is likely that the mutational screening technique used will miss a minority of mutations, particularly of single base substitutions and large genomic rearrangements, and therefore the numbers reported may be underestimates. Nevertheless, the results suggest that *ATM* mutations are unlikely to account for a substantial proportion of patients with dose-limiting complications of radiotherapy (although a small contribution cannot be excluded). These results are consistent with previous reports of three AT heterozygotes who had radiotherapy for breast cancer without unusual reactions (Ramsay et al, 1996; Fitzgerald et al, 1997) and 16 breast cancer cases showing radiotherapy complications in whom *ATM* mutations were not detected (Appleby et al, 1997).

From studies of relatives of AT patients, there is evidence that AT heterozygosity may be associated with an increased frequency of certain types of cancer, particularly breast carcinoma (Swift et al, 1987, 1991; Pippard et al, 1988). Additional evidence supporting this hypothesis has recently been obtained by genetic linkage analyses of families of AT cases using markers in the vicinity of *ATM* on chromosome 11q (Athma et al, 1996). However, direct examination by mutational screening of the *ATM* gene revealed mutations in 2/401 women with breast cancer compared with 2/202 control subjects (Fitzgerald et al, 1997). Whereas these data do not exclude a role for *ATM* as a low-penetrance breast cancer susceptibility gene (Bishop and Hopper, 1997), they do not lend strong support either. Although the present study is not a formal test of this hypothesis because there is no matched control group and the numbers are small, detection of a single AT heterozygote in 80 breast cancer cases does not add further weight to the notion that *ATM* is a low-penetrance breast cancer susceptibility gene.

Radiotherapy-induced breast shrinkage and distortion changes in a proportion of women after radiotherapy are progressive, permanent and of clinical relevance to the patient. They are also clearly related to radiotherapy dose. In the clinical trial from which these patients are drawn, a 10% difference in randomized dose (42.9 Gy in 13 fractions vs 39 Gy in 13 fractions) was associated with roughly a twofold difference in the chance of breast shrinkage (Owen et al, 1994). It has been shown in this study that testing for AT heterozygosity does not appear to offer a worthwhile approach for the identification of the radiosensitive subgroup of breast cancer patients and the search for the genetic loci responsible should continue.

© Cancer Research Campaign 1998

**Table 3** Oligonucleotide primers for amplification of individual *ATM* exons

| Exon | Nucleotide sequence (5′–3′) | Size (bp) | Nucleotide sequence (5′–3′) |
|---|---|---|---|
| 2 | CTAGCCCTTTTTTTGATTGGC | 310 | TGCTCATTCACTGATAGATGCA |
| 3 | CCTTTGACCAGAATGTGCCT | 371 | ATCTCGAATCAGGCGCTTAA |
| 4 | ATCTGCTTATCTGCTGCCGT | 385 | ATGCCAAATTCATATGCAAGG |
| 5 | GCTCTTTGTGATGGCATGAA | 303 | AAAAAAAAAAAAAACTCACGCG |
| 6 | AGTAGTTGCCATTCCAAGTGTC | 345 | AACTGTCAGGTCACTTGGGG |
| 7 | CTGCGACCTGGCTCTTAAAC | 469 | ATGGTCTTGCAAGATCAAAAGT |
| 8 | AGTTTGTACAGTTTGTTCCCCC | 414 | ATCAACCAGAGAAATCCAGAGG |
| 9 | GGTGTCTTCTAACGCTGATGC | 342 | CCCAAAATGCCCAGTTTAAA |
| 10 | GATACGAGATCGTGCTGTTCC | 350 | GGATTCCACTGAAAGTTTTCTG |
| 11 | TGTTAATGTGATGGAATAGTTT | 501 | AATGATCAGGGATATGTGAGTG |
| 12 | AAAGTCTTTGCCCCTCCAAT | 339 | AAATAAAGCCATCTGGCATCA |
| 13 | TTCTTTACATGGCTTTTGGTCT | 238 | TAAGATGCAGCTACTACCCAGC |
| 14 | TCAAAGTCCGAAGAAGAGAAGC | 497 | CCACCATCCTTGCTGTTTTT |
| 15 | AGCTATCCAGGATATGCCACC | 499 | TGCATGCTCTGCATCATGTA |
| 16 | TAAAAAGCAATACTAAACTA | 280 | CCAGGAGGTCAAGGCTACAATG |
| 17 | TCTGCCGAGAATAATTGTTTTT | 248 | TGTTGTGAGATGCATCCTTATT |
| 18 | TGACTACAGCATGCTCCTGC | 306 | CAATGAGGCCTCTTATACTGCC |
| 19 | ATATGGCTGTTGTGCCCTTC | 347 | TCAAAGACACCATGTGATTCTT |
| 20 | CGGCCTATGTTTATATACTT | 226 | GCTTAACAGAACACATCAGTT |
| 21 | TGTTCTTGAACTTCTGAAACCA | 346 | TGCATTCGTATCCACAGATAGC |
| 22 | GCAAGGTGAGTATGTTGGCA | 349 | TCAGCCTACGGGAAAAGAAC |
| 23 | GAATGGCCCTAGTAAATTGCC | 335 | TCTACTGCCATCTGCAGCAT |
| 24 | ATGCTTTGGAAAGTAGGGTTTG | 250 | TATGGGATATTCATAGCAAGCA |
| 25 | AAAAATGTGGAGTTCAGTTGGG | 349 | TGCCACTCAGAAAATCTAGCTT |
| 26 | TGTGTCAGATACTGTGCCAGTT | 434 | GTTGCTGGTGAGGGGACTT |
| 27 | GCTGATGGTATTAAAACAGTTT | 396 | GTTATATCTCATATCATTCAGG |
| 28 | TGCCTTTTGAGCTGTCTTGA | 339 | ATTACCTCAATTCAAAGGTGGC |
| 29 | AAATGGTTTTTGAATTTGGGG | 452 | GTGTCACGAGATTCTGTTCTCA |
| 30 | GTTTATTTTCTAGGATTCCTATC | 299 | TATGTTATTTACCTTTGGTTGA |
| 31 | ATGCTGAACAAAAGGACTTCTG | 487 | TGGACTACCTCTCCACTTCAGC |
| 32 | TTCGCAACGTTATGGTGGTAT | 525 | CAGGCTGGTCTTGAACTCC |
| 33 | TTTCACAGGCTTAACCAATACG | 249 | TCCCAAAATATTCTTTCCAAAA |
| 34 | CAAAAAGTGTTGTCTTCATGCT | 203 | TATGTGATCCGCAGTTGACTG |
| 35 | TTGACAACATTGGTGTGTAACG | 234 | GCCACATCCCCCTATGTTAA |
| 36 | ATGTATGATCTCTTACCTATGA | 315 | GCTTTAGTTACTGAGAATATCT |
| 37 | TTTGAAATTTTTTCAGTGGAGG | 304 | TTTAACAGTCATGACCCACAGC |
| 38 | GGAAAGGTACAATGATTTCCAC | 350 | AACAACAGTTTGAGTGGGGG |
| 39 | CGGGGCATGAAAATTTTAAG | 336 | TGGGATTCCATCTTAAATCCA |
| 40 | CTGGGACTGAGGGGAGATA | 200 | CATGTTAAAATTCAGCCGATAGTT |
| 41 | GGGGAAATGTGGTTTTTGG | 350 | ACCCTTATTGAGACAATGCCA |
| 42 | CAGGAGCTTCCAAATAGTATGT | 225 | GGCATCTGTACAGTGTCTATAA |
| 43 | CAGTTCAAACTCGTGTTGTTTG | 350 | AGCTTTGGGTTTTACACACACA |
| 44 | GGAGCCAGATAGTTTGTATGGC | 345 | TCTGGCTGTGTAAATATCCACC |
| 45 | TCTCTGGTTTTCTGTTGATATC | 270 | CAGTTGTTGTTTAGAATGAGGA |
| 46 | TTTGTCCTTTGGTGAAGCTATT | 238 | TTCAGAAAAGAAGCCATGACA |
| 47 | ATTTCCCTGAAAACCTCTTCTT | 227 | GGTAACAGAAAAGCTGCACTTT |
| 48 | CCGCATAGCATTTTTGTAGGT | 500 | CCTCAGGCTTTCTGTTTTTTAA |
| 49 | GGTAGNTGCTGCTTTCATTATT | 362 | TTGCTAATTTCAAGGCTCTAAT |
| 50 | GGGCAGTTGGGTACAGTCAT | 344 | GTAACAATGTTTCACTCCACCC |
| 51 | CGTGGGTTGGACAAGTTTG | 492 | TAAGCCGACCTTTAGAGCTCC |
| 52 | TTTCCCTGGGATAAAAACCC | 401 | TACACGATTCCTGACATCAAGG |
| 53 | CCACTTGTGCTAATAGAGGAGC | 320 | TTCCATTTCTTAGAGGGAATGG |
| 54 | TGCAGGCATACACGCTCTAC | 402 | CCAGCCTTGAACCGATTTTA |
| 55 | AAAGGCACCTAAGTCATTGACG | 489 | GGGAATGTTGAAGCCATCAG |
| 56 | CTTGACCTTCAATGCTGTTCC | 249 | TGCCAATATTTAGCCAATTTTG |
| 57 | CACATCGCATTTGTTTCTCTG | 340 | CAAAATCCCAAATAAAGCAGAA |
| 58 | ATTGGTTTGAGTGCCCTTTG | 299 | ATTATGAATATGGGCATGAGCC |
| 59 | AGGTCAACGGATCATCAAATG | 285 | AGCTGTCAGCTTTAATAAGCCA |
| 60 | ATCCTGTTCATCTTTATTGCCC | 339 | CAAAAATAAAACCTGCCAAACA |
| 61 | CTCAACATGGCCGGTTATG | 282 | CAAACAACATTCCATGATGACC |
| 62 | TGAGGAAGGCAGCCAGAG | 350 | GTGCAAAGAACCATGCCC |
| 63 | TTGACAACATTGGTGTGTAACG | 234 | GCCACATCCCCCTATGTTAA |
| 64 | TCCCCCATCAACTACCATGT | 324 | GAACAGTTTAAAGGCCTTGGG |
| 65 | CAAGGCCTTTAAACTGTTCACC | 309 | TTGGCAGGTTAAAAATAAAGGC |

© Cancer Research Campaign 1998

**Table 4** Summary of the *AT* sequence variants detected. Numbering is according to the cDNA sequence deposited in Genbank as U33841. Intronic variants are described as ± the number of nucleotides from the nearest exonic base in the cDNA sequence

| Exon (E)/intron (I) | Location | AA change | No. of heterozygotes out of the 41 cases | No. of heterozygotes out of the 39 controls |
|---|---|---|---|---|
| I2 | 160 –5 insT | None | 1 | 0 |
| E3 | A 201 G | Val 3 Val | 15 | 21 |
| I3 | 261–41 insAA | None | 11 | 14 |
| E4 | C 335 G | Ser 48 Cys | 3 | 1 |
| E8 | 898 insA | Stop at codon 251 | 1 | 0 |
| E8 | C 924 T | Val 244 Val | 1 | 1 |
| I13 | T 2088 –56 G | None | 1 | 1 |
| I13 | G 2088 –39 T | None | 0 | 1 |
| I15 | G 2438 +80 A | None | 1 | 0 |
| E18 | T 2761 C | Phe 857 Leu | 1 | 1 |
| I19 | 3027 +28 insA | None | 0 | 1 |
| I21 | T 3267 –80 C | None | 12 | 16 |
| E23 | C 3350 G | Pro 1053 Arg | 4 | 4 |
| I23 | 3473 –13 delT | None | 6 | 5 |
| E27 | G 4108 A | Gly 1306 Arg | 1 | 0 |
| E31 | C 4767 T | Pro 1525 Pro | 4 | 1 |
| I37 | T 5686 –8 C | None | 5 | 6 |
| E38 | G 5746 A | Asp 1852 Asn | 2 | 2 |
| E40 | T 5982 C | Ala 1930 Ala | 0 | 1 |
| E40 | G 6010 C | Val 1940 Leu | 3 | 1 |
| I47 | 6997 –57 insATT | None | 12 | 19 |
| E49 | G 7251 A | Ala 2353 Ala | 0 | 1 |
| E51 | G 7572 A | Arg 2460 His | 0 | 1 |
| E52 | C 7710 T | Ala 2506 Ala | 1 | 0 |
| E59 | C 8683 T | Arg 2830 His | 0 | 1 |
| I62 | A 9039 +60 G | None | 10 | 17 |
| E64 | C 9389 G | None (3′ untranslated) | 0 | 1 |

### ACKNOWLEDGEMENTS

We would like to thank women with breast cancer who participated in this study for their support and encouragement. The work was supported by a Radiation Protection Research Programme Grant (A3.8/RRX 43) from the Department of Health and by the Cancer Research Campaign. We would also like to acknowledge the help of Mr S. Ebbs and Dr John Peacock.

### REFERENCES

Appleby JM. Barber JBP. Levine E. Varley JM. Taylor AMR. Stankovic T. Heighway J. Warren C and Scott D (1997) Absence of mutations in the ATM gene in breast cancer patients with severe responses to radiotherapy. *Br J Cancer* **76**: 1546–1549

Athma P. Rappaport R and Swift M (1996) Molecular genotyping shows that ataxia telangiectasia heterozygotes are predisposed to breast cancer. *Cancer Genet Cytogenet* **92**: 130–134

Bishop DT and Hopper J (1997) AT-tributable risks? *Nature Genet* **15/3**: 226

Boder E and Sedgwick RP (1958) Ataxia telangiectasia: a familial syndrome of progressive cerebellar ataxia, oculocutaneous telangiectasia and frequent pulmonary infection. *Pediatrics* **21**: 526–554

Byrd PJ. McConville CM. Cooper P. Parkhill J. Stankovic T. McGuire GM. Thick JA and Taylor MR (1996) Mutations revealed by sequencing the 5′ half of the gene for the ataxia telangiectasia. *Hum Mol Genet* **5**: 145–149

Easton DF (1994) Cancer risks in AT heterozygotes. *Int J Radiat Biol* **66** (suppl. 6): s177–182

FitzGerald MG. Bean JM. Hedge SR. Unsal H. MacDonald DJ. Harkin DP. Finkelstein DM. Isselbacher KJ and Haber DA (1997) Heterozygous ATM mutations do not contribute to early onset of breast cancer. *Nature Genet* **15**: 307–310

Ganguly A. Rock MJ and Prockop DJ (1993) Conformation-sensitive gel electrophoresis for rapid detection of single-base differences in double-stranded PCR products and DNA fragments. *Proc Natl Acad Sci USA* **90**: 10325–10329

Gilad S. Khosravi R. Shkedy D. Uziel T. Ziv Y. Savitsky K. Rotman G. Smith S. Chessa L. Jorgensen TJ. Harnik R. Frydman M. Sanal O. Portnoi S. Goldwicz Z. Jaspers NGJ. Gatti RA. Lenoir G. Lavin MF. Tatsumi K. Wegner RD. Shiloh Y and Barshira A (1996) Predominance of null mutations in ataxia telangiectasia. *Hum Mol Genet* **5**: 433–439

Gotoff SP. Amirnokri E and Liebner EJ (1967) Ataxia telangiectasia neoplasia. untoward response to x-radiation and tuberous sclerosis. *Am J Dis Child* **114**: 617–627

Johansen J. Bentzen SM and Overgaard J (1996) Relationship between the in-vitro radiosensitivity of skin fibroblasts and the expression of subcutaneous fibrosis. telangiectasia. and skin erythema after radiotherapy. *Radiother Oncol* **40**: 101–109

Jorgensen TJ and Shiloh Y (1996) The ATM gene and the radiobiology of ataxia telangiectasia. *Int J Radiat Biol* **69**: 527–537

Maher Committee (1995) *Management of Adverse Effects Following Breast Radiotherapy*. London: Royal College of Radiologists

Nagasawa H. Kraemer KH. Shiloh Y and Little JB (1987) Detection of ataxia telangiectasia heterozygous cell lines by post-irradiation cumulative labelling index: measurements with coded samples. *Cancer Res* **47**: 398–402

Owen JR. Yarnold JR. Bliss JM. Ebbs SR. Regan J. Harrington G and Ashton A (1996) RT fractionation sensitivity: proposals for a UK national trial. *Radiother Oncol* Suppl. 1: 572

Pippard EC. Hall AJ. Barker DJP and Bridges BA (1988) Cancer in homozygotes and heterozygotes of ataxia telangiectasia and xeroderma pigmentosum in Britain. *Cancer Res* **48**: 2929–2932

Ramsey J. Birrell G. Lavin M (1996) Breast cancer and radiotherapy in ataxia telangiectasia heterozygote. *Lancet* **347**: 1627

Savitsky K. Bar-Shira A. Gilad S. Rothman G. Ziv Y. Vanagaite L. Tagle DA. Smith S. Uziel D. Skez S. Ashkenazi M. Pecker I. Frydman M. Harnik R. Patanjali SR. Simmons A. Clines GA. Sartiel A. Gatti RA. Chessa L. Sanal O. Lavin MF. Jaspers NGT. Taylor AMR. Arlett CF. Miki T. Weissman SM. Lovett M. Collins FS and Shiloh Y (1995) A single ataxia telangiectasia gene with a product similar to PI-3 kinase. *Science* **268**: 1749–1753

Swift M. Reitnauer PJ. Morrell D and Chase CL (1987) Breast and other cancers in families with ataxia telangiectasia. *New Engl J Med* **316**: 1289–1294

Swift M. Morrell D. Massey RB and Chase CL (1991) Incidence of cancer in 161 families affected by ataxia telangiectasia. *New Engl J Med* **325**: 1831–1836

© Cancer Research Campaign 1998

Taylor AM, Harnden DG, Arlett CF, Harcourt SA, Lehmann AR, Stevens S and Bridges BA (1975) Ataxia telangiectasia: a human mutation with abnormal radiation sensitivity. *Nature* **258**(5534): 427–429

Tucker SL, Turesson I and Thames HD (1992) Evidence for individual differences in the radiosensitivity of human skin. *Eur J Cancer* **28A**: 1283–1291

Turesson I (1989) The progression rate of late radiation effects in normal tissue and its impact on dose-response relationships. *Radiotherapy* **15**: 217–226

Turesson I (1990) Individual variation and dose dependency in the progression rate of skin telangiectasia. *Int J Radiat Oncol Biol Phys* **19**: 1569–1574

Weeks DE, Paterson MC, Lange K, Andrais B, Davies RC, Yoder F and Gatti RA (1991) Assessment of chronic radiosensitivity as an in vitro assay for identification of ataxia telangiectasia. *Radiat Res* **128**: 90–99

West CM, Elyan SA, Berry P, Cowan R and Scott D (1995) A comparison of the radiosensitivity of lymphcytes from normal donors, cancer patients, individuals with ataxia telangiectasia (*AT*) and AT heterozygotes. *Int J Radiat Biol* **68**(2): 197–203

© Cancer Research Campaign 1998